# US Population Size and Outcomes of Adults on Liver Transplant Waiting Lists

**DOI:** 10.1001/jamanetworkopen.2025.1759

**Published:** 2025-03-25

**Authors:** Tomohiro Tanaka, George Wehby, Mark Vander Weg, Keith Mueller, David Axelrod

**Affiliations:** 1 Division of Gastroenterology and Hepatology, University of Iowa Carver College of Medicine, Iowa City; 2 Department of Health Management and Policy, College of Public Health, University of Iowa, Iowa City; 3 Center for Access & Delivery Research and Evaluation, Iowa City Veterans Affairs Health Care System, Iowa City, Iowa; 4 Department of Economics, University of Iowa, Iowa City; 5 National Bureau of Economic Research, Cambridge, Massachusetts; 6 Department of Community and Behavioral Health, College of Public Health, University of Iowa, Iowa City; 7 Department of Surgery, University of Iowa Carver College of Medicine, Iowa City

## Abstract

**Importance:**

Disparities in organ supply and demand led to geographic inequities in the score-based liver transplant (LT) allocation system, prompting a change to allocation based on acuity circles (AC) defined by fixed distances. However, fixed distances do not ensure equivalent population size, potentially creating new sources of disparity.

**Objective:**

To estimate the association between population size around LT centers and waiting list outcomes for critically ill patients with chronic end-stage liver disease and high Model for End-stage Liver Disease (MELD) scores or acute liver failure (ALF).

**Design, Setting, and Participants:**

This US nationwide retrospective cohort study included adult (aged ≥18 years) candidates for deceased donor LT wait-listed between June 18, 2013, and May 31, 2023. Follow-up was completed June 30, 2023. Participants were divided into pre-AC and post-AC groups.

**Exposure:**

Population size within defined radii around each LT center (150 nautical miles [nm] for participants with high MELD scores and 500 nm for those with ALF) based on AC allocation policy.

**Main Outcomes and Measures:**

LT candidate waiting list mortality and dropout rate were analyzed using generalized linear mixed-effect models with random intercepts for center and listing date before and after AC implementation. Fine-Gray competing risk regression, accounting for clustering, was used as a secondary model.

**Results:**

The study analyzed 6142 LT candidates (1581 with ALF and 4561 with high MELD scores) during the pre-AC era and 4344 candidates (749 with ALF and 3595 with high- MELD scores) in the post-AC era, for a total of 10 486 participants (6331 male [60.5%]; mean [SD] age, 48.5 [7.1] years). In the high-MELD cohort, being listed at a center in the lowest tertile of population size was associated with increased waiting list mortality in the AC era (adjusted odds ratio [AOR], 1.68; 95% CI, 1.14-2.46). Doubling of the population size was associated with a 34% reduction in the odds of mortality or dropout (AOR, 0.66; 95% CI, 0.49-0.90). These results were consistent with those of the extended Fine-Gray models and were also corroborated by multiple sensitivity analyses. However, there were no significant population density–associated disparities in the ALF cohort.

**Conclusions and Relevance:**

In this retrospective nationwide cohort study, being wait-listed in less populated regions was associated with greater mortality among critically ill LT candidates with high MELD scores, underscoring the limitations of allocation systems based purely on fixed distances.

## Key Points

**Question:**

Is the population size from which donor organs can be allocated associated with liver transplant waiting list outcomes for critically ill candidates in the US?

**Findings:**

In a cohort study of 10 486 adult candidates for deceased donor liver transplants, those listed at centers in areas with smaller populations had higher waiting list mortality rates in the post–acuity circle era compared with those in more populated areas. Critically ill patients with high Model for End-Stage Liver Disease scores had 1.7 times higher odds of mortality or dropout at low-density centers, a disparity not observed in patients with acute liver failure.

**Meaning:**

These findings highlight the need for policy adjustments to ensure equitable access to donor organs across areas with different population sizes.

## Teaser

This cohort study estimates the association between population size within the acuity circle of liver transplant centers and waiting list outcomes for patients with acute liver failure or chronic end-stage liver disease.

## Introduction

Organ allocation policies for liver transplants (LT) in the US have been repeatedly revised to improve equity and minimize waiting list deaths.^1^ The Organ Procurement and Transplantation Network (OPTN) introduced the acuity circle (AC)–based allocation policies in February 2020. Previously, LTs were allocated within OPTN-designated regional boundaries, resulting in disparate median Model for End-Stage Liver Disease (MELD) scores and different outcomes across 11 regions.^2,3^ The AC policy replaced the region-based system with concentric circles measured in nautical miles (nm) from donor hospitals to equitably distribute organs and reduce waiting list inconsistencies.^4^ This method aims to balance organ availability and minimize variations in illness severity (measured by MELD scores) at the time of transplant. Despite its goals, the AC policy faced criticism for not considering important metrics such as population density and/or size.^5^ Incorporating population density and size could address geographic disparities more effectively, as donor populations can vary substantially (eg, 150 nm around Philadelphia includes the entire mid-Atlantic region while the same circle around Salt Lake City contains mostly minimally populated regions).^5^ Further, variability in donation rates across organ procurement organizations complicates donor availability within circles.^6,7,8^ Simulations suggested that larger circles can impose logistical challenges while smaller circles (≤150 nm) can exacerbate geographic disparities due to unequal population sizes.^5,9^

Prior to the AC policy, known as the Share 35 rule,^10^ LT candidates with acute liver failure (ALF) or chronic end-stage liver disease (ESLD) and a MELD score of 35 or higher had designated OPTN region-wide access to organs, bypassing center-specific factors such as population size. Following the implementation of the AC policy, however, the impact of patient-level disparities due to population size variations around LT centers has not been evaluated. This is crucial, as patients with severe liver dysfunction face high mortality without an LT.^11,12^ This group includes patients with ALF (status 1A in the UNet organ transplant Web platform), for whom the initial allocation (first match run) targets LT centers within a 500-nm radius. Similarly, candidates with chronic ESLD and MELD scores of 37 or higher have their first match run targeting centers within a 150-nm radius.^13^

Therefore, we conducted a nationwide retrospective cohort study to estimate the association between population size within the AC of the LT center and patient-level LT waiting list outcomes for patients with ALF (listed as status 1A) or those with chronic ESLD and a MELD score of 37 or higher at the time of listing. Our research hypothesis posited that LT centers located in areas with smaller populations experience higher rates of waiting list mortality or dropout due to clinical deterioration prior to organ availability.

## Methods

### Data Sources

Data for LT candidates were extracted from the OPTN Standard Transplant Analysis and Research (STAR) file. For the regional analysis, population and latitude and longitude of each transplant center zip code were identified from interactive maps (Simplemaps; Pareto Software LLC). The study protocol was determined not human participants research and exempt from the requirement for informed consent and was approved by the University of Iowa Institutional Review Board. This study followed the Strengthening the Reporting of Observational Studies in Epidemiology (STROBE) reporting guideline.

### Study Design and Population

This study used a nationwide retrospective cohort design to evaluate the association between population size within the AC of LT centers and clinical outcomes for LT waiting list candidates in the US. The cohort included adult (aged ≥18 years) first-time wait-listed candidates for deceased donor LT, encompassing listings from the initiation of the Share 35 policy on June 18, 2013,^14^ through May 31, 2023, and followed up until June 30, 2023. Race and ethnicity were documented by organ transplant coordinators during organ donor assessments and by transplant program staff at the time of patient waiting list registration. Categories for race and ethnicity included American Indian or Alaska Native, Asian, Black, Hispanic, Native Hawaiian or Other Pacific Islander, White, and multiracial. Given the altered allocation policy for centers in Hawaii and Puerto Rico and extending the distribution range to 2400 nm in Hawaii and 1100 nm in Puerto Rico to enhance access for urgent candidates in these remote locations,^15^ we excluded them from the cohort. Based on the date of listing, participants were divided into 2 groups: the pre-AC era (until February 3, 2020) and the post-AC era (on or after February 4, 2020). We stratified the cohort rather than using an interaction term for the unstratified cohort, allowing covariate outcomes to vary by era for a less restrictive assessment of the AC policy’s modification on population density and/or size. Patients were followed up from the date of listing to death, delisting, LT, loss to follow-up, or end of the study period. Participants in the pre-AC group who survived through February 4, 2020, were censored, ensuring waiting list outcomes were equivalently measured in both eras. We then stratified the population into 2 distinct cohorts based on their listing status: individuals listed with a MELD score of 37 or higher (high-MELD cohort) and those with ALF designated as status 1A (ALF cohort). We chose a cutoff of 37 for the high-MELD cohort to include all candidates with chronic ESLD who were most prioritized under both the AC and the Share 35 policies. We provided a comprehensive description of the participants, encompassing their demographic and clinical characteristics, as well as the outcomes on LT waiting lists.

### Measures of Exposures, Outcomes, and Covariates

The primary exposure of interest in this study is the population size within the AC surrounding LT centers. For the high-MELD cohort, a radius of 150 nm was defined around each LT center, and for the ALF cohort, a radius of 500 nm was defined around each LT center. The total population count within these radii was calculated and defined as the population size within each center’s AC. These radii were chosen based on the distance used for the first match run in the AC policy.^13^ Geographic information system software was used to calculate the centroid for each zip code, which was then included in the AC population size calculation if it fell within the AC boundary. Candidates were grouped into tertiles of population size around the LT centers where they were wait-listed, classified as low population size (low-PS), middle population size (middle-PS), and high population size (high-PS) tiers. Among the 31 candidates listed at multiple centers (0.6%), the center with the highest population size was designated as the listing center. It is important to note that while AC policy is based on circles around the donor hospital, the donor population accessible to a patient is based on the population surrounding the transplant center. Primary outcomes were waiting list outcomes (receiving LT, removal due to death or clinical deterioration, and removal due to clinical improvement). Secondary outcomes included donor age, use of donation after circulatory death, geographic distance from the donor hospital to transplant center, and posttransplant death hazard. We collected demographic, clinical, center, and area-level (ie, each AC) covariates. Area-level covariates included the Herfindahl-Hirschman Index (HHI),^16^ quantifying the concentration of LT centers.

### Statistical Analysis

For patient demographic characteristics, continuous variables were compared using the *t* test while categorical variables were analyzed using the χ^2^ test. For patients with chronic ESLD and a high MELD score or ALF, time waiting is minimal as patients receive an organ, die, or occasionally recover sufficiently to be removed (in the case of ALF). Given this brief turnover, a two-way generalized linear mixed-effect model (GLMM) for a binary outcome with a logit link function was used as our primary model to evaluate the association between population size around the LT center and waiting list outcome in both pre-AC (control) and post-AC periods.^17^ Middle-PS areas served as the reference group. The GLMM modeled removal from LT waiting list due to death or clinical deterioration within 30 days as the outcome of interest and was adjusted for covariates recorded at listing, including demographic factors (sex, age, race and ethnicity), clinical factors (MELD-sodium score, moderate to severe ascites, grades 3-4 encephalopathy, mechanical ventilation, hemodialysis), center activity (mean number of yearly transplant cases), and competition within the ACs (HHI [log-transformed]). The GLMM included LT center–level and listing date–level random intercepts. Results were presented as adjusted odds ratios (AORs) of death or dropout on the waiting list. In addition, to account for censoring, semiparametric competing-risk survival models by Fine and Gray,^18^ extended to accommodate correlation within clusters (of LT center and date of listing) as proposed by Zhou et al,^19^ were used as secondary models. These models treated death and dropout due to clinical deterioration on the LT waiting list as the event of interest, with LT as the competing risk, and adjusted for the same covariates as the GLMM. We chose this approach over the Cox proportional hazards regression model with frailty terms, which may lead to biased results in the presence of substantial and related competing risks^20,21^; in this cohort, 60% to 80% of candidates received LT, a rate that can render estimates of hazards of the event of interest (waiting list mortality) from Cox proportional hazards regression models unstable and biased. The Figure shows a directed acyclic graph (DAG)^23^ to inform theory-based variable selection for these models. This DAG was drawn with software that provides a graphic user interface tailored to draw and analyze causal diagrams (DAGitty; R Platform).^22^ We also visualized the unadjusted cumulative incidence of waiting list mortality and dropout, with receiving LT as a competing risk, stratified by pre-AC and post-AC population size groups. Last, we conducted 6 additional sensitivity and supplemental analyses to ensure the robustness of our main models, the details of which are presented in eMethods 1 in Supplement 1. Transplant-related outcomes among those who underwent LT, including proportion of marginal grafts (donor age continuous and >65 years), donation after circulatory death, travel distance greater than 150 nm for the high-MELD and greater than 500 nm for the ALF cohorts, patient survival, and failure events categorized as death) are presented in eMethods 2 in Supplement 1.

**Figure.**
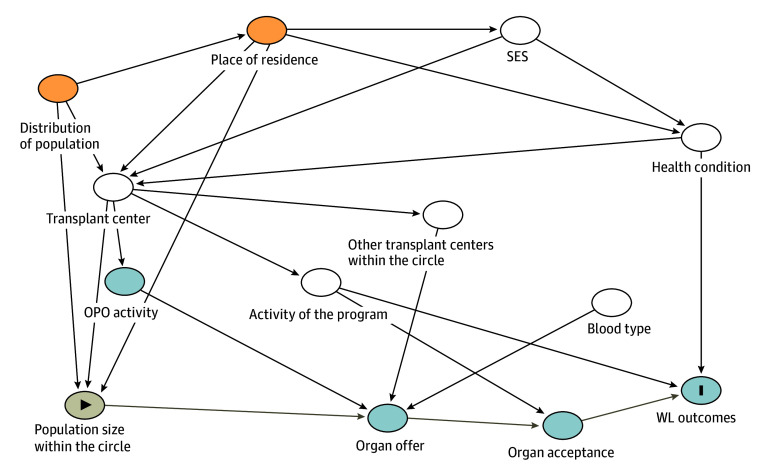
Directed Acyclic Graph (DAG) to Inform Theory-Based Variable Selection for Study Models The exposure (population size within the respective acuity circle), outcomes (death or dropout on the waiting list [WL]), and other covariates in this study are illustrated. An arrow (edge) represents a possible association. For DAGs, the selection of variables (nodes) and the direction of edges are guided by a theory-based approach grounded in empirical literature. The arrows represent adjusted paths between variables. This DAG was drawn receiwith DAGitty, which provides a graphic user interface tailored to draw and analyze causal diagrams.^22^ OPO indicates organ procurement organization; SES, socioeconomic status. The directional symbol in the population size circle indicates the exposure of interest, and the line symbol in the WL outcomes circle indicates the outcomes of interest.

Statistical significance was based on 2-sided tests with *P* < .05. All analyses were performed using R statistical software, version 4.3.2 (R Program for Statistical Computing).

## Results

### Baseline Characteristics

The study included 6142 LT candidates, consisting of 4561 with high MELD scores and 1581 with acute liver failure (ALF), from the pre-AC era, and 4344 candidates, consisting of 3595 with high MELD scores and 749 with ALF, from the post-AC era. The study population therefore included 10 486 LT candidates, 4141 (39.5%) female and 6345 (60.5%) male with a mean (SD) age of 48.5 (7.1) years. In terms of race and ethnicity, 523 (5.0%) participants were Asian; 1047 (10.0%), Black; 1835 (17.5%), Hispanic; 6096 (58.1%), White; and 985 (9.4%), other (including American Indian or Alaska Native, Native Hawaiian or Other Pacific Islander, and multiracial). Table 1 and Table 2 summarize demographic, clinical, and socioeconomic factors across patient subgroups based on the population size around the LT center in the pre-AC and post-AC eras. The mean age at waitlisting was similar, but gender and ethnicity varied: the high-MELD cohort had more male participants, while the ALF cohort had more female. There were no distinct patterns in the racial distribution across cohorts. Socioeconomic factors varied significantly, with higher neighborhood income in middle-PS areas for the high-MELD cohort. Lower educational attainment was more common in the low-PS group, while Medicaid coverage was highest in the high-PS group.

**Table 1.** Patient Demographic Data for High-MELD Cohort in Eras Before and After AC

Characteristic	Pre-AC era				Post-AC era			
	Middle-PS center (n = 1456)	Low-PS center (n = 1582)	High-PS center (n = 1523)	*P* value	Middle-PS center (n = 1237)	Low-PS center (n = 1245)	High-PS center (n = 1113)	*P* value
Age at listing, mean (SD), y	51.4 (11.4)	50.9 (11.3)	51.7 (11.4)	.17	48.2 (11.6)	48.2 (11.6)	48.4 (11.9)	.79
Gender, No. (%)								
Female	534 (36.7)	577 (36.5)	580 (38.1)	.58	455 (36.8)	435 (34.9)	445 (40.0)	.04
Male	922 (63.3)	1005 (63.5)	943 (61.9)		782 (63.2)	810 (65.1)	668 (60.0)	
Ethnicity and race (%)								
Asian	64 (4.4)	57 (3.6)	83 (5.4)	<.001	44 (3.6)	30 (2.4)	60 (5.4)	<.001
Black	179 (12.3)	163 (10.3)	163 (10.7)		115 (9.3)	95 (7.6)	100 (9.0)	
Hispanic	156 (10.7)	254 (16.1)	444 (29.2)		142 (11.5)	209 (16.8)	312 (28.0)	
White	1036 (71.2)	1056 (66.8)	817 (53.6)		922 (74.5)	853 (68.5)	636 (57.1)	
Other^a^	21 (1.4)	52 (3.3)	16 (1.1)		14 (1.1)	58 (4.7)	5 (0.4)	
Etiology								
AH	77 (5.3)	70 (4.4)	110 (7.2)	<.001	128 (10.3)	109 (8.8)	129 (11.6)	<.001
ALF-cirrhosis	535 (36.7)	565 (35.7)	647 (42.5)		450 (36.4)	450 (36.1)	488 (43.8)	
Autoimmune	68 (4.7)	69 (4.4)	45 (3.0)		27 (2.2)	27 (2.2)	28 (2.5)	
HBV	49 (3.4)	39 (2.5)	48 (3.2)		32 (2.6)	24 (1.9)	10 (0.9)	
HCV	220 (15.1)	235 (14.9)	226 (14.8)		50 (4.0)	34 (2.7)	29 (2.6)	
Cancer	23 (1.6)	38 (2.4)	15 (1.0)		12 (1.0)	12 (1.0)	11 (1.0)	
MASH	184 (12.6)	215 (13.6)	181 (11.9)		149 (12.0)	157 (12.6)	99 (8.9)	
PBC	26 (1.8)	21 (1.3)	27 (1.8)		8 (0.6)	16 (1.3)	13 (1.2)	
PSC	36 (2.5)	42 (2.7)	27 (1.8)		33 (2.7)	15 (1.2)	12 (1.1)	
Other	238 (16.3)	288 (18.2)	197 (12.9)		348 (28.1)	401 (32.2)	294 (26.4)	
BMI at listing, mean (SD)	31.1 (7.0)	30.8 (6.9)	30.0 (7.0)	<.001	30.4 (7.1)	224.3 (6717.8)	29.8 (6.9)	.38
Calculated MELD at listing, mean (SD)^b^	41.2 (3.7)	41.1 (3.8)	41.1 (3.5)	.54	41.2 (3.8)	41.0 (3.6)	41.1 (3.5)	.57
Assigned MELD at listing, mean (SD)^b^	39.2 (2.9)	39.2 (3.0)	39.3 (2.9)	.27	39.1 (2.6)	39.2 (3.0)	39.3 (3.0)	.39
OPTN region, No. (%)								
1	115 (7.9)	0	68 (4.5)	<.001	102 (8.2)	0	49 (4.4)	<.001
2	118 (8.1)	0	530 (34.8)		57 (4.6)	0)	391 (35.1)	
3	243 (16.7)	309 (19.5)	0		261 (21.1)	216 (17.3)	0	
4	46 (3.2)	481 (30.4)	0		24 (1.9)	436 (35.0)	0	
5	185 (12.7)	193 (12.2)	703 (46.2)		130 (10.5)	115 (9.2)	447 (40.2)	
6	0	133 (8.4)	0		0	89 (7.1)	0	
7	344 (23.6)	103 (6.5)	8 (0.5)		207 (16.7)	79 (6.3)	46 (4.1)	
8	0	194 (12.3)	0		0	159 (12.8)	0	
9	0	46 (2.9)	214 (14.1)		0	54 (4.3)	180 (16.2)	
10	239 (16.4)	0	0		210 (17.0)	0	0	
11	166 (11.4)	123 (7.8)	0		246 (19.9)	97 (7.8)	0	
Hemodialysis at listing, No. (%)	705 (48.4)	716 (45.3)	879 (57.7)	<.001	671 (54.2)	651 (52.3)	649 (58.3)	.01
Encephalopathy, No. (%)	429 (29.5)	481 (30.4)	372 (24.4)	<.001	356 (28.8)	404 (32.4)	276 (24.8)	<.001
Mechanical ventilator at listing, No. (%)	205 (14.1)	245 (15.5)	219 (14.4)	.51	135 (10.9)	76 (6.1)	104 (9.3)	<.001
Ascites, No. (%)	795 (54.6)	845 (53.4)	893 (58.6)	.009	644 (52.1)	692 (55.6)	579 (52.0)	.13
Days on the waiting list, mean (SD)	11.3 (166.3)	7.2 (51.1)	6.5 (39.4)	.48	9.7 (10.1)	8.3 (17.0)	9.1 (19.3)	.14
Neighborhood income, mean (SD), US $	75 579.4 (31 828.9)	66 501.7 (24 651.6)	67 577.7 (37 279.2)	<.001	79 535.9 (29 812.4)	68 764.4 (24 530.2)	69 131.0 (40 041.9)	<.001
Medicare payer, No. (%)	325 (22.3)	285 (18.0)	512 (33.6)	<.001	313 (25.3)	265 (21.3)	385 (34.6)	<.001
Educational level high school or lower, No. (%)	779 (53.5)	713 (45.1)	869 (57.1)	<.001	565 (45.7)	513 (41.2)	532 (47.8)	.004
Blood group, No. (%)								
O	643 (44.2)	794 (50.2)	736 (48.3)	.01	547 (44.2)	610 (49.0)	530 (47.6)	.31
A	585 (40.2)	564 (35.7)	530 (34.8)		481 (38.9)	447 (35.9)	420 (37.7)	
B	182 (12.5)	173 (10.9)	201 (13.2)		161 (13.0)	144 (11.6)	122 (11.0)	
AB	46 (3.2)	51 (3.2)	56 (3.7)		48 (3.9)	44 (3.5)	41 (3.7)	

Abbreviations: AC, acuity circle; AH, alcohol-associated hepatitis; ALF, acute liver failure; BMI, body mass index (calculated as the weight in kilograms divided by the height in meters squared); HBV, hepatitis B virus; HCV, hepatitis C virus; MASH, metabolic dysfunction-associated steatohepatitis; MELD, Model for End-Stage Disease; OPTN, Organ Procurement and Transplantation Network; PBC, primary biliary cirrhosis; PS, population size; PSC, primary sclerosing cholangitis.

^a^ Includes American Indian or Alaska Native, Native Hawaiian or Other Pacific Islander, and multiracial.

^b^ Scores range from 6 to 40, with 35 or greater considered OPTN access to transplant.

**Table 2.** Patient Demographic Data for ALF Cohort in Eras Before and After AC

Characteristic	Pre-AC era				Post-AC era			
	Middle-PS center (n = 448)	Low-PS center (n = 522)	High-PS center (n = 611)	*P* value	Middle-PS center (n = 247)	Low-PS center (n = 212)	High-PS center (n = 290)	*P* value
Age at listing, mean (SD), y	40.8 (15.8)	43.6 (15.4)	43.3 (15.5)	.01	42.5 (15.7)	42.3 (16.3)	45.2 (16.2)	.07
Gender, No. (%)								
Female	306 (68.3)	316 (60.5)	381 (62.4)	.03	161 (65.2)	150 (70.8)	176 (60.7)	.07
Male	142 (31.7)	206 (39.5)	230 (37.6)		86 (34.8)	62 (29.2)	114 (39.3)	
Ethnicity and race, No. (%)								
Asian	26 (5.8)	79 (15.1)	46 (7.5)	<.001	17 (6.9)	27 (12.7)	19 (6.6)	<.001
Black	110 (24.6)	44 (8.4)	140 (22.9)		61 (24.7)	22 (10.4)	99 (34.1)	
Hispanic	44 (9.8)	131 (25.1)	42 (6.9)		30 (12.1)	62 (29.2)	28 (9.7)	
White	257 (57.4)	250 (47.9)	374 (61.2)		130 (52.6)	92 (43.4)	142 (49.0)	
Other^a^	11 (2.4)	18 (3.5)	9 (1.5)		9 (3.7)	9 (4.2)	2 (0.7)	
Acetaminophen etiology, No. (%)	95 (21.2)	63 (12.1)	100 (16.4)	.001	45 (18.2)	33 (15.6)	39 (13.4)	.22
BMI at listing, mean (SD)	29.0 (7.0)	32.0 (66.3)	28.9 (7.2)	.35	29.1 (8.8)	29.5 (6.9)	28.6 (6.8)	.47
Calculated MELD score at listing, mean (SD)^b^	34.8 (8.2)	34.9 (8.3)	36.1 (8.5)	.02	37.5 (8.5)	36.9 (8.4)	36.4 (9.2)	.37
OPTN region, No. (%)								
1	73 (16.3)	0	0	<.001	39 (15.8)	0	0	<.001
2	0	0	228 (37.3)		0	0	101 (34.8)	
3	109 (24.3)	43 (8.2)	0		49 (19.8)	25 (11.8)	0	
4	59 (13.2)	80 (15.3)	0		20 (8.1)	28 (13.2)	0	
5	12 (2.7)	310 (59.4)	0		15 (6.1)	123 (58.0)	0	
6	0	46 (8.8)	0		0	16 (7.5)	0	
7	128 (28.6)	18 (3.4)	0		71 (28.7)	5 (2.4)	0	
8	28 (6.2)	25 (4.8)	24 (3.9)		17 (6.9)	15 (7.1)	10 (3.4)	
9	26 (5.8)	0	149 (24.4)		24 (9.7)	0	75 (25.9)	
10	0	0	102 (16.7)		0	0	52 (17.9)	
11	13 (2.9)	0	108 (17.7)		12 (4.9)	0	52 (17.9)	
Hemodialysis at listing, No. (%)	75 (16.7)	100 (19.2)	74 (12.1)	.004	62 (25.1)	67 (31.6)	40 (13.8)	<.001
Encephalopathy, No. (%)	269 (60.0)	289 (55.4)	343 (56.1)	.29	132 (53.4)	124 (58.5)	151 (52.1)	.34
Mechanical ventilator at listing, No. (%)	215 (48.0)	234 (44.8)	273 (44.7)	.51	97 (39.3)	70 (33.0)	120 (41.4)	.15
Ascites, No. (%)	59 (13.2)	80 (15.3)	95 (15.5)	.51	43 (17.4)	40 (18.9)	41 (14.1)	.34
Neighborhood income, mean (SD), US $	70 955.4 (27 521.8)	797 82.8 (32 000.3)	62 778.6 (40 967.4)	<.001	84 394.6 (52 183.9)	79 222.8 (30 070.8)	62 534.4 (41 783.2)	<.001
Medicare payer, No. (%)	93 (20.8)	127 (24.3)	150 (24.5)	.29	54 (21.9)	57 (27.0)	93 (32.1)	.03
Educational level high school or lower, No. (%)	243 (54.2)	244 (46.7)	316 (51.7)	.06	130 (52.6)	95 (44.8)	150 (51.7)	.19
Blood type, No. (%)								
O	208 (46.4)	265 (50.8)	300 (49.1)	.46	130 (52.6)	101 (47.6)	148 (51.0)	.81
A	163 (36.4)	167 (32.0)	198 (32.4)		76 (30.8)	75 (35.4)	91 (31.4)	
B	55 (12.3)	71 (13.6)	92 (15.1)		28 (11.3)	25 (11.8)	40 (13.8)	
AB	22 (4.9)	19 (3.6)	21 (3.4)		13 (5.3)	11 (5.2)	11 (3.8)	

Abbreviations: AC, acuity circle; BMI, body mass index (calculated as the weight in kilograms divided by the height in meters squared); ALF, acute liver failure; MELD, Model for End-Stage Disease; OPTN, Organ Procurement and Transplantation Network; PS, population size.

^a^ Includes American Indian or Alaska Native, Native Hawaiian or Other Pacific Islander, and multiracial.

^b^ Scores range from 6 to 40, with 35 or greater considered OPTN access to transplant.

### Waiting List and Post-LT Outcomes

#### Crude Waiting List Outcomes

The 30-day crude waiting list outcomes (receipt of LT, death or dropout, spontaneous recovery, and remaining on the waiting list at day 30 following being wait-listed) are summarized in Table 3. In short, in the high-MELD cohort, LT rates were generally high in the pre-AC era (range, 1138 of 1523 [74.7%] for high-PS center to 1205 of 1582 [76.2%] for low-PS center) and improved in the post-AC era (range, 1012 of 1245 [81.3%] for low-PS to 1058 of 1237 [85.5%] for middle-PS; *P* < .001). Death or dropout rates due to clinical deterioration also improved in the post-AC era (range, 329 of 1582 [20.8%] for low-PS to 338 of 1523 [22.2%] for high-PS pre AC vs 150 of 1237 [12.1%] for middle-PS to 205 of 1245 [16.5%] for low-PS post AC; P<.001), but with variability by population size within ACs. Rates of spontaneous recovery were low (<1% for all population sizes and both AC eras). In contrast, the ALF cohort had lower transplant rates (range, 260 of 448 [58.0%] for middle-PS [pre-AC era] to 149 of 212 [70.3%] for low-PS [post-AC era]), lower death or dropout rates (range, 29 of 212 [13.7%] for low-PS [post-AC era] to 95 of 448 [21.2%] for middle-PS [pre-AC era]), and higher rates of spontaneous recovery (24 of 212 [11.8%] for low-PS [post-AC era] to 75 of 448 [16.7%] for middle-PS [pre-AC era]) across eras than those with chronic ESLD. Of note, a minority remained on the waiting list 30 days after being wait-listed (17 [2.2%] in the high-MELD cohort and 104 [4.5%] in the ALF cohort).

**Table 3.** Crude 30-Day Waiting List Outcomes in the Pre-AC and Post-AC Eras Stratified by Population Size Group

Outcome	Pre-AC era				Post-AC era			
	Middle-PS center	Low-PS center	High-PS center	*P* value	Middle-PS center	Low-PS center	High-PS center	*P* value
High-MELD cohort, No.	1456	1582	1523		1237	1245	1113	
Waiting list outcome, No. (%)^a^								
LT	1099 (75.5)	1205 (76.2)	1138 (74.7)	.79	1058 (85.5)	1012 (81.3)	906 (81.4)	.045
Death or dropout	320 (22.0)	329 (20.8)	338 (22.2		150 (12.1)	205 (16.5)	180 (16.2)	
Recovered	10 (0.7)	11 (0.7)	13 (0.9)		4 (0.3)	4 (0.3)	4 (0.4)	
On waiting list	27 (1.9)	37 (2.3)	35 (2.2)		25 (1.9)	24 (1.9)	23 (2.1)	
ALF cohort, No.	448	522	611		247	212	290	
Waiting list outcome, No. (%)^b^								
LT	260 (58.0)	338 (64.8)	382 (62.5)	.22	163 (66.0)	149 (70.3)	182 (62.8)	.51
Death or dropout	95 (21.2)	83 (15.9)	113 (18.5)		40 (16.2)	29 (13.7)	49 (16.9)	
Recovered	75 (16.7)	79 (15.1)	88 (14.4)		31 (12.6)	24 (11.3)	46 (15.9)	
On waiting list	18 (4.0)	22 (4.2)	28 (4.6)		13 (5.3)	10 (4.7)	13 (4.5)	

Abbreviations: AC, acuity circle; ALF, acute liver failure; LT, liver transplant; MELD, Model for End-Stage Disease; PS, population size.

^a^ For all candidates in pre-AC vs post-AC, *P* < .001.

^b^ For all candidates in pre-AC vs post-AC, *P* = .09.

#### Result of the Primary and Secondary Models

The outcomes of the primary (GLMM) and secondary (extended Fine-Gray) models for waiting list outcomes are shown in Table 4. For the high-MELD cohort compared with recipients listed in the middle-PS centers, candidates in the low-PS group had higher odds of pretransplant mortality in the post-AC era (AOR, 1.68; 95% CI, 1.14-2.46) but not in the pre-AC era (AOR, 1.20; 95% CI, 0.84-1.71). Those in the high-PS group showed no significant advantages compared with the middle-PS group either in the pre-AC (AOR, 1.18; 95% CI, 0.71-1.98) or post-AC period (AOR, 0.77; 95% CI, 0.42-1.40). Use of the extended Fine-Gray regression model found that the low-PS group was associated with an increase in hazard of death or dropout due to clinical deterioration in the post-AC period (subdistribution hazard ratio [SHR], 1.63; 95% CI, 1.28-2.01). No significant differences were observed in the pre-AC period. Waiting list mortality or dropout rate in the high-PS group did not show a significant difference compared with the middle-PS group, either in the pre-AC (SHR, 0.71; 95% CI, 0.49-1.01) or post-AC era (SHR, 0.84; 95% CI, 0.62-1.15), although the hazard for death or dropout was decreased in both.

**Table 4.** Results of Primary and Secondary Model Analysis

Model	GLMM, AOR (95% CI)	Fine-Gray, SHR (95% CI)
**High MELD**		
Pre-AC		
Low-PS	1.20 (0.84-1.71)	1.07 (0.91-1.27)
High-PS	1.18 (0.71-1.98)	0.71 (0.49-1.01)
Post-AC		
Low-PS	1.68 (1.14-2.46)	1.63 (1.28-2.01)
High-PS	0.77 (0.42-1.40)	0.84 (0.61-1.15)
**ALF**		
Pre-AC		
Low-PS	0.92 (0.52-1.62)	0.93 (0.57-1.52)
High-PS	0.58 (0.34-0.99)	0.74 (0.48-1.14)
Post-AC		
Low-PS	1.32 (0.48-3.64)	1.06 (0.50-2.26)
High-PS	1.11 (0.46-2.67)	1.01 (0.48-2.12)

Abbreviations: AC, acuity circle; ALF, acute liver failure; AOR, adjusted odds ratio; GLMM, generalized linear mixed-effect model; MELD, Model for End-Stage Disease; PS, population size; SHR, subdistribution hazard ratio (of death or dropout due to clinical deterioration).

In the pre-AC era, ALF candidates in the high-PS group showed a lower hazard of waiting list mortality or dropout compared with the middle-PS group in the GLMM (AOR, 0.58; 95% CI, 0.34-0.99) and exhibited similar findings in Fine-Gray models (SHR, 0.74; 95% CI, 0.48-1.14). However, in the post-AC era, candidates in the low-PS or the high-PS group did not show a significantly different risk of waiting list mortality or dropout compared with the middle-PS group (AOR for low-PS group, 1.32 [95% CI, 0.48-3.64] and AOR for the high PS group, 1.11 [95% CI, 0.46-2.67]). Likewise, the Fine-Gray competing risk survival regression did not show significant differences in waiting list mortality or dropout due to clinical deterioration in the post-AC era among those in either the low-PS (SHR, 1.06; 95% CI, 0.50-2.26) or high-PS (SHR, 1.01; 95% CI, 0.48-2.12) groups. The unadjusted cumulative incidence curves for transplant and death or dropout, with the other as a competing risk, stratified by PS tertiles in both the pre-AC and post-AC eras in the 2 cohorts, are illustrated in the eFigure in Supplement 1.

#### Results of the Supplementary and Sensitivity Analyses for Waiting List Mortality and Transplant-Related Measures

Last, the results of the 6 supplemental and sensitivity analyses for waiting list outcomes are presented in eTable 1 in Supplement 1 and were essentially consistent with those of the main models, including the analysis using log base 2-transformed PS as a continuous variable for the surrogate exposure of interest, which remained consistent with the primary models in the high MELD cohort in the post-AC era (AOR, 0.66; 95% CI, 0.49–0.90), indicating that each PS doubling reduces the odds of death/dropout by 34% on the LT waiting list. Results of analysis of transplant-related outcomes are also shown in eTable 2 in Supplement 1.

## Discussion

This cohort study found that mortality and dropout rates of severely ill LT candidates decreased overall following AC implementation, although the extent of improvement varied based on the population size within the AC of an LT center. LT candidates with chronic ESLD (MELD score ≥37) who were listed in low-PS LT centers had 1.7 times higher odds of death or dropout due to clinical deterioration compared with those in medium-PS centers following the implementation of the AC distribution policy. This association was observed across multiple sensitivity analyses. We identified that candidates listed in low-PS centers were significantly less likely to receive an organ during the first match run from surrounding donor hospitals within 150 nm. However, among patients wait-listed with ALF, for whom a 500-nm radius is used for the first donor match, a low-PS center within this radius was not associated with disparities in access in the post-AC era and may have mitigated the overprioritization of high-PS centers observed in the pre-AC era.

Our finding of disparities associated with PS around each LT center in the waiting list outcomes for patients with high MELD scores and chronic ESLD using a 150-nm circle for allocation adds to previous reports of a wide range and large variance in ratios of liver supply to demand within 150-nm fixed-distance circles in the US LT allocation system.^5,9^ We postulate that lower PS within the 150-nm circle reduces the availability of allografts contributing an uneven supply-demand ratio,^5^ leading to higher waiting list mortality. This finding was consistent after accounting for factors such as local competition for donors (HHI) and the activity levels of transplant centers (as indicated by the number of transplant cases during the study period). For the ALF cohort served by a 500-nm circle, there were no associations with population size in the post-AC era, which is consistent with a previous report suggesting that larger geographic circles effectively reduce variation in supply-demand ratios for this group.^9^ Notably, the trend of overprioritization of those in high-PS areas within the radius diminished in the post-AC era. Although logistical barriers inherent in larger sharing regions are not neglibable,^24^ the broad span of the 500-nm circle likely facilitates a more balanced organ allocation, smoothing over regional differences that smaller circles might exacerbate.

One contribution of our study is providing insights into the distribution of liver allografts among severely ill patients with ESLD and identifying notable disparities associated with population size around the LT centers, which were exacerbated in the post-AC era. To our knowledge, this is the first study to delineate such disparities in patient-level outcomes on the waiting list, potentially offering useful information for policy-making processes. Nevertheless, alongside efforts to equitably distribute organs, enhancing the organ supply is crucial. This includes increasing deceased donation rates, optimizing organ procurement organization performance, and improving marginal organ use by transplant centers using novel preservation techniques.^9,25,26^

### Limitations

This study has some limitations. The retrospective design and reliance on the OPTN STAR file may introduce residual confounding. The AC policy’s impact may be confounded by the COVID-19 pandemic, although sensitivity analyses across postpandemic years yielded consistent, robust estimates, supporting the reliability of our findings. The relatively small sample size led to wide CIs in some of our models (especially for the ALF cohort), limiting the precision of certain estimates. The study’s generalizability may be limited to patients without ALF or those with lower MELD scores at listing. Modeling the impact of allocation circle size and heterogeneity is challenging due to variable waiting list dynamics. Importantly, patients in this study were categorized based on MELD scores at listing and did not include patients with initially low MELD scores but subsequent deterioration. Additionally, the OPTN STAR file includes only wait-listed candidates, leaving the impact of population size on non–wait-listed patients with ESLD and broader disparities in the LT process unaddressed.^27^

## Conclusions

This cohort study highlights notable disparities faced by critically ill candidates with chronic ESLD in less populated areas in the US when allocation is based on straight geographic distance rather than donor organ potential. The increased risk of waiting list mortality and dropout underscores the need for policy adjustments to promote equity in LT allocations and ensure access for populations outside metropolitan areas. The analysis shows that patients with ALF who have access within a 500-nm circle are not disadvantaged, unlike patients with chronic ESLD and high MELD scores within a 150-nm circle. This distinction emphasizes the importance of defining AC size and incorporating population size and/or density into the allocation scheme, considering the economic and logistical burdens of broader circles. The findings highlight the need to address geographic disparities by including population density and size in the proposed continuous distribution allocation policy revision.^28^

## Notes

Supplement 1. **eMethods 1.** Additional Analyses for Waitlist Outcomes **eMethods 2.** Transplant-Related Outcomes **eTable 1.** Results of the Sensitivity Analyses **eTable 2.** Hazards of Death Post Transplant Across Subgroups Using Cox Proportional Hazard Models **eFigure.** Analysis of Transplant-Related Outcomes

Supplement 2.
Data Sharing Statement
