# Supplementary material for: US Population Size and Outcomes of Adults on Liver Transplant Waiting Lists
Source: JAMA Netw Open. 2025 Mar 25;8(3):e251759. doi: 10.1001/jamanetworkopen.2025.1759 (PMC11937946; doi:10.1001/jamanetworkopen.2025.1759)
Supplement: Supplement 1. — eMethods 1. Additional Analyses for Waitlist Outcomes eMethods 2. Transplant-Related Outcomes eTable 1. Results of the Sensitivity Analyses eTable 2. Hazards of Death Post Transplant Across Subgroups Using Cox Proportional Hazard Models eFigure. Analysis of Transplant-Related Outcomes [file jamanetwopen-e251759-s001.pdf]

Supplementary Online Content

Tanaka T, Wehby G, Vander Weg M, Mueller K, Axelrod D. US population size and outcomes of adults on liver transplant waitlists. *JAMA Netw Open.* 2025;8(3):e251759. doi:10.1001/jamanetworkopen.2025.1759

eMethods 1. Additional Analyses for Waitlist Outcomes

eMethods 2. Transplant-Related Outcomes

eTable 1. Results of the Sensitivity Analyses

eTable 2. Hazards of Death Post Transplant Across Subgroups Using Cox Proportional Hazard Models

eFigure. Analysis of Transplant-Related Outcomes

This supplementary material has been provided by the authors to give readers additional information about their work.

© 2025 Tanaka T et al. *JAMA Network Open*.

eMethods 1. Additional Analyses for Waitlist Outcomes

We conducted six distinct supplemental analyses and sensitivity checks for the models evaluating waitlist outcomes; (i) First, although our initial intention was to test the hypothesis that lower, middle, and high-population size (PS) rank in their association with the risk of mortality, we included (a) PS within the acuity circles (AC) as a continuous variable, transformed using the base-2 logarithm due to right skewness, and (b) a binary variable comparing the Low-PS group to the others (Mid-PS and High-PS) in the primary GLMM. (ii) Second, given the high recovery rate on the waitlist in acute liver failure (ALF) groups (18%), we evaluated a three-category outcome (waitlist mortality, recovery, or receiving liver transplant [LT]) using multinomial logistic regression, excluding random intercepts but including Organ Procurement and Transplantation Network (OPTN)-Region fixed-effects. (iii) Third, we re-estimated the primary models adding centers in Hawaii and Puerto Rico to ensure the robustness of our nationally representative estimates. (iv) Fourth, given Herfindahl-Hirschman Indexes (HHIs) are endogenous to population size, we re-estimated the regression models excluding these covariates. (v) Fifth, to account for the unmeasured confounding impact of the COVID-19 pandemic, we ran the GLMM excluding the period from February 4 to May 8, 2020 (per OPTN). Additionally, we analyzed the divided cohorts of candidates listed during (1) June 1, 2020, to May 31, 2021, (2) June 1, 2021, to May 31, 2022, and (3) June 1, 2022, to May 31, 2023. (vi) Finally, we evaluated the interaction term (Post-AC = 1 * Low/High PS) in the GLMM, inclusive of both pre- and post-AC periods.

The results of these analyses are summarized in Table S1. The results of the log base 2-transformed PS and the binary exposure variable of Lowest-PS vs. others, used as alternative exposures of interest, were consistent with the primary models for patients with high Model for End-Stage Liver Disease (MELD) scores in the post-AC era (aOR: 0.66; 95% CI: 0.49–0.90, and 1.65; 1.12-2.42, respectively). This indicates that a log unit increase in PS (i.e., a doubling of PS) is associated with a 34% reduction in the odds of death/dropout due to deterioration on LT waitlist. By contrast, among the ALF cohort in the post-AC era, PS, when treated as a continuous (logarithmically scaled) variable was not significantly associated with waitlist mortality/dropout, which is also consistent with the primary models for this cohort. For the ALF cohort, the multinomial logistic regression showed no significant association between population size and the outcome, which is consistent overall with the primary model. Lastly, results from the GLMM for the analyses including centers in Hawaii and Puerto Rico, excluding HHI from the regression models, and re-defining the post-AC era accounting for the impact of COVID-19 pandemic were overall consistent with the primary models.

© 2025 Tanaka T et al. *JAMA Network Open*.

eMethods 2. Transplant-Related Outcomes

For transplant-related outcomes among those who received an LT, we described the proportion of marginal grafts (Donor age [continuous and >65 years], donation after circulatory death (DCD), and travel distance >150/500 nm for the High-MELD/ALF cohorts). A Cox proportional hazards model with center-level frailty term assessed overall patient survival post-transplantation, with failure events categorized as death. Data were censored at the most recent follow-up.

The results of these sensitivity analyses are summarized in Table S2. The analysis of donor-related outcomes suggests that the Low-PS group had a higher chance of receiving a donor from beyond 150 miles for High MELD patients (74% vs. 64.5% vs. 53.3% in low, middle, and high-PS groups, respectively, $p<0.001$) and beyond 500 miles for the ALF cohort (61.8% vs. 48.1% vs. 38.5% in low, middle, and high-PS groups, respectively, $p<0.001$). The proportion of patients receiving livers from donation after circulatory death (DCD)- donors was low (1-2%) in both the High MELD and ALF cohorts, regardless of the PS-based group, and was not statistically significant between eras. Post-transplant patient survival for High MELD and ALF cohorts was comparable across each PS -based group across eras.

© 2025 Tanaka T et al. *JAMA Network Open*.

eTable 1. Results of the Sensitivity Analyses

: (i) Population size (PS) within acuity circles (AC) was included as the natural log in the primary GLMM. (ii) Multinomial logistic regression assessed a three-category outcome (waitlist mortality, recovery, LT) with OPTN-Region fixed effects. (iii) Models were re-estimated, including Hawaii and Puerto Rico. (iv) Regressions excluded HHIs to address endogeneity with PS. (v) Models excluded data from February 4 to May 8, 2020, for COVID-19 impact and analyzed cohorts listed during three sequential periods from June 1, 2020, to May 31, 2023. (vi) Estimates of the beta coefficients of the interaction terms (Post-AC * High/Low-PS) in the GLMM, inclusive of both pre- and post-AC periods.

(i)		
High MELD:		**GLMM (aOR, 95% CI)**
Pre-AC	Population size (PS)*	1.04 (0.81-1.34)
	Low-PS vs. higher	1.04 (0.81-1.34)
Post-AC	PS *	0.66 (0.49-0.90)
	Low-PS vs. higher	1.65 (1.12-2.42)
ALF:		**GLMM (aOR, 95% CI)**
Pre-AC	PS *	0.83 (0.43-2.07)
	Low-PS vs. higher	1.32 (0.77–2.27)
Post-AC	PS*	2.04 (0.66-6.25)
	Low-PS vs. higher	1.20 (0.49-2.93)
(ii)		
ALF:		
		**Multinomial logistic regression (RRR, 95% CI)**
Pre-AC	Low-PS:	1.20 (0.84-1.71)
	High-PS:	1.18 (0.71-1.99)
Post-AC	Low-PS:	1.68 (0.94-2.46)
	High-PS:	0.77 (0.42-1.40)
(iii)		
High MELD:		**GLMM (aOR, 95% CI)**
Pre-AC	Low-PS:	1.21 (0.98-1.45)
	High-PS:	1.03 (0.76-1.39)
Post-AC	Low-PS:	1.89 (1.36-263)
	High-PS:	0.94 (0.64-1.39)
ALF:		**GLMM (aOR, 95% CI)**
Pre-AC	Low-PS:	1.08 (0.60 - 1.94)
	High-PS:	0.60 (0.35 - 1.02)
Post-AC	Low-PS:	0.90 (0.32 - 2.52)

© 2025 Tanaka T et al. *JAMA Network Open*.

	High-PS:	1.23 (0.53 - 2.88)
(iv)		
High MELD:		**GLMM (aOR, 95% CI)**
Pre-AC	Low-PS:	1.22 (0.88-1.79)
	High-PS:	1.10 (0.69-1.75)
Post-AC	Low-PS:	1.65 (1.05-2.58)
	High-PS:	0.79 (0.40-1.55)
ALF:		**GLMM (aOR, 95% CI)**
Pre-AC	Low-PS:	0.89 (0.45-1.76)
	High-PS:	0.67 (0.29-1.54)
Post-AC	Low-PS:	1.50 (0.55-4.08)
	High-PS:	1.22 (0.51-2.91)
(v)		
High MELD:		**GLMM (aOR, 95% CI)**
Pre-AC	Low-PS:	1.18 (0.81-1.72)
	High-PS:	1.25 (0.77-2.02)
Post-AC	Low-PS:	1.69 (1.11-2.60) [5/9/2020 -]<br>1.60 (0.95, 2.71) [6/2020-5/2023]<br>1.77(1.03,3.05) [6/2021-5/2023]<br>1.88(1.06,3.34) [6/2022-5/2023]
	High-PS:	0.72 (0.37-1.42)
ALF:		**GLMM (aOR, 95% CI)**
Pre-AC	Low-PS:	0.85 (0.42-1.73)
	High-PS:	0.59 (0.28-1.24)
Post-AC	Low-PS:	1.35 (0.49-3.72)
	High-PS:	1.09 (0.43-2.75) [5/9/2020 -]<br>1.08 (0.23, 5.10) [6/2020-5/2023]<br>1.09 (0.19, 6.41) [6/2021-5/2023]<br>1.10 (0.17, 7.06) [6/2022-5/2023]
(vi)		**β (SE), p value**
High MELD	Low-PS:	0.44 (0.16), p=0.005
	High-PS:	0.30 (0.19), p=0.11
ALF	Low-PS:	0.49 (0.32), p=0.13
	High-PS:	0.38 (0.30), p=0.21

*Unit of the variable is persons within the AC, and log-base2 transformed.

© 2025 Tanaka T et al. *JAMA Network Open*.

eTable 2. Hazards of Death Post Transplant Across Subgroups Using Cox Proportional Hazard Models

Era	Cohort	Population Size	Hazard Ratio (95% CI)
Pre-AC	High-MELD	Highest tertile	0.99 (0.76-1.27)
Pre-AC	High-MELD	Lowest tertile	1.12 (0.91-1.38)
Post-AC	High-MELD	Highest tertile	0.94 (0.61-1.46)
Post-AC	High-MELD	Lowest tertile	0.89 (0.60-1.30)
Pre-AC	ALF	Highest tertile	1.15 (0.49-2.96)
Pre-AC	ALF	Lowest tertile	1.24 (0.56-2.74)
Post-AC	ALF	Highest tertile	2.48 (0.33-5.34)
Post-AC	ALF	Lowest tertile	1.75 (0.57-5.34)

© 2025 Tanaka T et al. *JAMA Network Open*.

eFigure. Analysis of Transplant-Related Outcomes

Cumulative incidence (unadjusted) of transplant with death/dropout as a competing risk (dashed line) and death/dropout with transplant as a competing risk (solid line) stratified by population size (PS) within the respective acuity circle for (A) High- Model for End-Stage Liver Disease (MELD) cohort pre-Acuity Circle (AC) [Gray's test, p=0.29 and 0.27, respectively], (B) High-MELD cohort post-AC [p=0.02 and 0.008, respectively] (C) Acute liver failure (ALF) cohort pre-AC [p=0.31 and 0.38, respectively] and (D) ALF cohort post-AC [p=0.22 and 0.17, respectively]. The y-axis for solid lines represents survival probability, and the dashed lines represent the cumulative incidence rate of liver transplant (LT). Note that most of the candidates in the ALF group who remained on the waitlist ended up with spontaneous recovery (91.8% in pre-AC era and 90.9% in post-AC era).

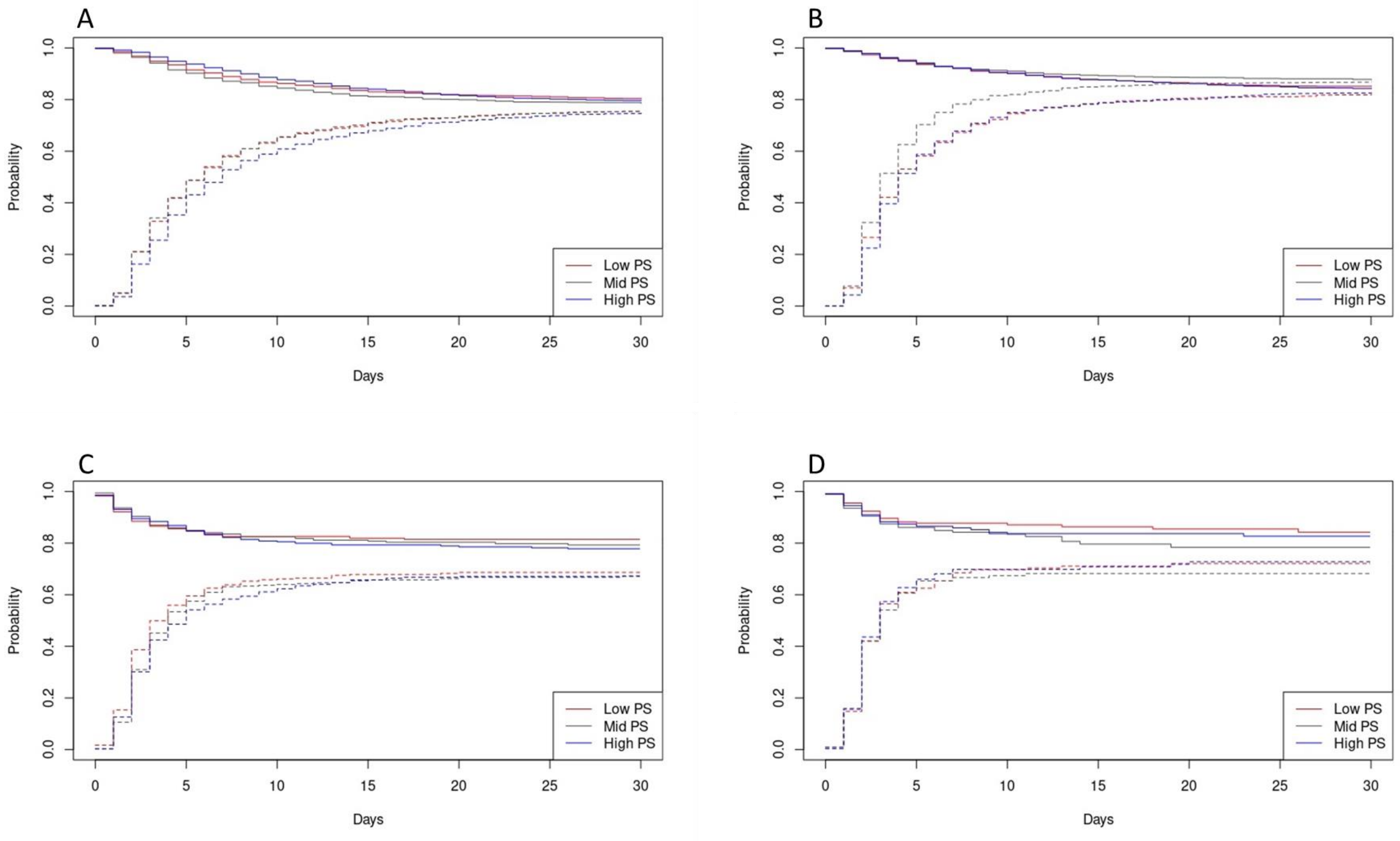

© 2025 Tanaka T et al. *JAMA Network Open*.
