# Supplementary material for: US Population Size and Outcomes of Adults on Liver Transplant Waiting Lists
Source: JAMA Netw Open. 2025 Mar 25;8(3):e251759. doi: 10.1001/jamanetworkopen.2025.1759 (PMC11937946; doi:10.1001/jamanetworkopen.2025.1759)
Supplement: Supplement 2. — Data Sharing Statement [file jamanetwopen-e251759-s002.pdf]

Data Sharing Statement

Tanaka. US Population Size and Outcomes of Adults on Liver Transplant Waiting Lists. *JAMA Netw Open*. Published March 25, 2025. doi:10.1001/jamanetworkopen.2025.1759

Data
Data available: No
